# Kinetic Analysis of the Slow Skeletal Myosin MHC-1 Isoform from Bovine Masseter Muscle

**DOI:** 10.1016/j.jmb.2007.08.050

**Published:** 2007-11-09

**Authors:** M.J. Bloemink, N. Adamek, C. Reggiani, M.A. Geeves

**Affiliations:** 1 Protein Science Group, Department of Biosciences, University of Kent, Canterbury, Kent CT2 7NJ, UK; 2 Department of Anatomy and Physiology, University of Padua, Via Marzolo 3, Padua, 35131 Italy

**Keywords:** MHC, myosin heavy chain, MLC, myosin light chain, RLC, regulatory light chain, ELC, essential light chain, S1, myosin subfragment S1, ^BM^S1, bovine masseter S1, ^PS^S1, pig soleus S1, ^BC^S1, bovine cardiac S1, ^RS^S1, rabbit soleus S1, TCA, trichloroacetic acid, subfragment 1, actin, ATPase, strain-sensor, ADP

## Abstract

Several heavy chain isoforms of class II myosins are found in muscle fibres and show a large variety of different mechanical activities. Fast myosins (myosin heavy chain (MHC)-II-2) contract at higher velocities than slow myosins (MHC-II-1, also known as β-myosin) and it has been well established that ADP binding to actomyosin is much tighter for MHC-II-1 than for MHC-II-2. Recently, we reported several other differences between MHC-II isoforms 1 and 2 of the rabbit. Isoform II-1 unlike II-2 gave biphasic dissociation of actomyosin by ATP, the ATP-cleavage step was significantly slower for MHC-II-1 and the slow isoforms showed the presence of multiple actomyosin–ADP complexes. These results are in contrast to published data on MHC-II-1 from bovine left ventricle muscle, which was more similar to the fast skeletal isoform. Bovine MHC-II-1 is the predominant isoform expressed in both the ventricular myocardium and slow skeletal muscle fibres such as the masseter and is an important source of reference work for cardiac muscle physiology. This work examines and extends the kinetics of bovine MHC-II-1. We confirm the primary findings from the work on rabbit soleus MHC-II-1. Of significance is that we show that the affinity of ADP for bovine masseter myosin in the absence of actin (represented by the dissociation constant *K*_D_) is weaker than originally described for bovine cardiac myosin and thus the thermodynamic coupling between ADP and actin binding to myosin is much smaller (*K*_AD_/*K*_D_ ∼ 5 instead of *K*_AD_/*K*_D_ ∼ 50). This may indicate a distinct type of mechanochemical coupling for this group of myosin motors. We also find that the ATP-hydrolysis rate is much slower for bovine MHC-II-1 (19 s^−1^) than reported previously (138 s^−1^). We discuss how this work fits into a broader characterisation of myosin motors from across the myosin family.

## Introduction

The myosin superfamily consists of at least 18 classes of ATP-dependent motor proteins that interact with actin filaments and are involved in a large variety of physiological processes, such as muscle contraction, phagocytosis, cell motility and vesicle transport.^1^^,^^2^ Class II myosins (found in muscle) consist of two heavy chains (MHC) and two pairs of light chains: the regulatory light chains (RLC) and essential light chains (ELC). The C termini of the myosin heavy chains dimerize and form a coiled-coil (“myosin tail”) whereas the N-termini form the two myosin “heads” or “motor-domains”. The most familiar activity of class II myosins is muscle contraction. All myosins appear to undergo the same ATP driven cycle of interaction of myosin with actin, known as the cross-bridge cycle, yet myosins show a wide variety of different mechanical activities. We are interested in the variety of different types of mechanochemical activities shown both within and between the members of the myosin family.

Myosins from fast contracting, anaerobic, muscle fibres are amongst the best characterized myosins with events of the biochemical cross-bridge cycle having been correlated with mechanical and structural data from contracting muscle fibres. These fast myosins comprise a subgroup of the myosins family known as MHC-II-2 with three primary isoforms found in the mammalian adult skeletal muscle, 2a, 2b and 2x, each expressed from a different gene.^3^ The isoform MHC-II-1 is found in slow, aerobic skeletal muscle fibres and in ventricular cardiac muscle tissue where it is known as MHC-β. The myosin light chains (MLC) also differ between myosins with muscle-specific isoforms of both light chains.^4^ Whereas a single regulatory light chain isoform (MLC2s) is found in both the slow skeletal muscle and in the ventricle of mammals that have been examined, there are two isoforms of slow muscle essential light chains. One is found predominantly in the adult slow skeletal and the ventricle muscle (called MLC-1s/v or MLC-1s/b). The other (MLC-1sa) predominates in smooth and non-muscle tissue and in some skeletal muscle during development.^5^ The MLC-1s/b is larger and includes and an N-terminal extension, rich in proline and lysine. Unlike molluscan and smooth muscle myosins, where light chain phosphorylation and calcium binding are the primary regulators of myosin's activity, the light chains of striated muscles seem to have relatively little effect on enzymatic activity of the isolated myosin head.^6^ However, the presence of specific light chain isoforms and their removal does have substantial effects on the generation of force and motility by striated myosin.^6^ The MLC stabilise the lever-arm or neck of the myosin head and they are in a position to modulate the translation of the isomerisation of the head into mechanical force or movement.^7^

Fast muscle fibres containing the MHC-II-2, as their name suggests, contract at higher velocities and are more susceptible to fatigue than slow muscle fibres containing MHC-II-1 and both properties have been related to the properties of the myosin heavy chain isoform expressed in the muscle. It is well established that ADP binding to actomyosin is much tighter for MHC-II-1 (slow isoform) than for MHC-II-2 (fast isoform).^8^ We recently examined the solution biochemical kinetic properties of the slow isoform MHC-II-1 from rabbit soleus muscle and compared it to the well characterised fast isoform MHC-II-2 from rabbit psoas muscle.^9^ This showed several significant differences between the two isoforms including:

(1) Unlike MHC-II-2, the MHC-II-1 gave biphasic dissociation of actomyosin by ATP.
(2) The tight binding of ADP to actomyosin was confirmed for MHC-II-I with at least three conformations of actomyosin–ADP present in equilibrium.
(3) The rate constant for ADP release from the predominant actomyosin–ADP complex was the correct size to limit the shortening velocity of MHC-II-1 containing soleus muscle fibres.
(4) MHC-II-1 has a significantly reduced rate constant for the ATP hydrolysis step.

Together these results indicate that the kinetic differences between the fast and slow MHC-II isoforms are greater than at first expected and that MHC-II-1 has much in common with some of the much slower smooth and non-muscle myosins (see Discussion).

Three of the above novel observations (numbers (1), (2) and (4)) were in contrast to published data on the same isoform isolated from bovine ventricle muscle. Bovine MHC-II-1 has been widely studied from cardiac ventricle tissue and is an important source of reference work for cardiac muscle physiology.^8^^,^^10^ It is therefore essential to test if the differences observed between the rabbit and bovine isoforms are due to the experimental differences or species differences. The previous studies on bovine cardiac S1 (^BC^S1) reported that the ATP-hydrolysis rate is much higher (*k*_+3_ + *k*_*-*3_ = 138 s^−1^)^11,12^ than the hydrolysis rate reported for rabbit soleus S1 under similar conditions (^RS^S1, *k*_+3_ + *k*_-3_ = 22 s^−1^).^9^ Also, unlike ^RS^S1, the ATP-induced dissociation of AM, in the presence or absence of ADP, could not be fitted unambiguously to two components.^10^ In order to address these differences we used bovine MHC-II-1 isolated from the slow, bovine masseter muscle. Bovine masseter muscle is large, easily accessible and contains >95% MHC-II-1, which makes it convenient for large scale biochemical preparations.^13^ This tissue is readily available and the isolated myosin and S1 are stable when stored at −20 °C. In order to further examine potential species differences we also isolated myosin S1 from pig soleus fibres as another source of MHC-II-1 myosin. This tissue is not as pure in myosin isoform content^14^ but does provide a second reference material. Masseter muscle fibres have a maximum shortening velocity (*V*_0_ = 0.27 μm s^−1^) of 40% of the value for rabbit soleus fibres (*V*_0_ = 0.67 μm s^−1^) at 12 °C^13^ whereas the pig soleus fibres are even slower (*V*_0_ = 0.17 μm s^−1^).^13^^,^^15^ Since *V*_0_ has been shown to be a property of the MHC isoform, the species differences in the isoforms used here are expected to be significant. The work presented here confirms the primary finding from the work of rabbit soleus, and extends these studies to slow bovine and porcine myosins. Of significance is that we show that the affinity of ADP for bovine masseter myosin is weaker than originally described for bovine cardiac myosin and thus the thermodynamic coupling between ADP and actin binding to myosin is much smaller^10^^,^^11^ (*K*_AD_/*K*_D_ ∼5 instead of *K*_AD_/*K*_D_ ∼ 50). This may indicate a distinct type of mechanochemical coupling for this group of myosin motors. We also find that the ATP-hydrolysis rate is much slower for ^BM^S1 (19 s^−1^) than reported previously for ^BC^S1 (138 s^−1^).

We discuss how this work fits into a broader characterization of myosin motors from across the myosin family. Indeed the relevance of our observations on the significant differences between fast and slow muscle myosin isoforms in the release of ADP has been underlined by a recent study by Iwamoto *et al*.,^16^ published as we submitted this work. They have shown, using a novel X-ray scattering approach, that slow muscle acto––S1 has a distinct movement of the lever arm on binding ADP, whereas little movement was detected with a fast muscle isoform. Larger movements were observed with the S1 from smooth and non-muscle myosin II isoforms. These observations are consistent with earlier electron micrograph images of ADP induced movements of the lever arm of smooth muscle^17^ and cytoplasmic myosin from the wider myosin family, such as myosin I^18^ and myosin VI.^19^ Thus, biochemical and structural studies are coming together to define how ADP release is a key event in defining the mechano-chemical coupling of different types of myosin.

## Results

### Nucleotide binding to ^BM^S1 in the absence of actin

**Figure 1:**
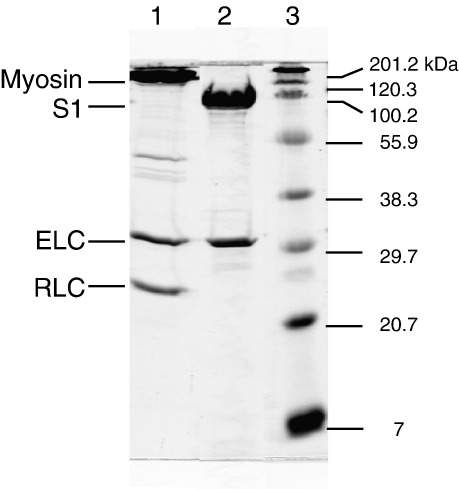
SDS–polyacrylamide electrophoresis gel of ^BM^S1 purification. Samples were run on a SDS–15% gel with glycine/Tris/SDS running buffer. Lane 1, bovine masseter myosin (^BM^Myosin) with two light chains. Lane 2, ^BM^S1 after chymotrypsin digestion and column purification. Lane 3, molecular mass marker.

**Figure 2:**
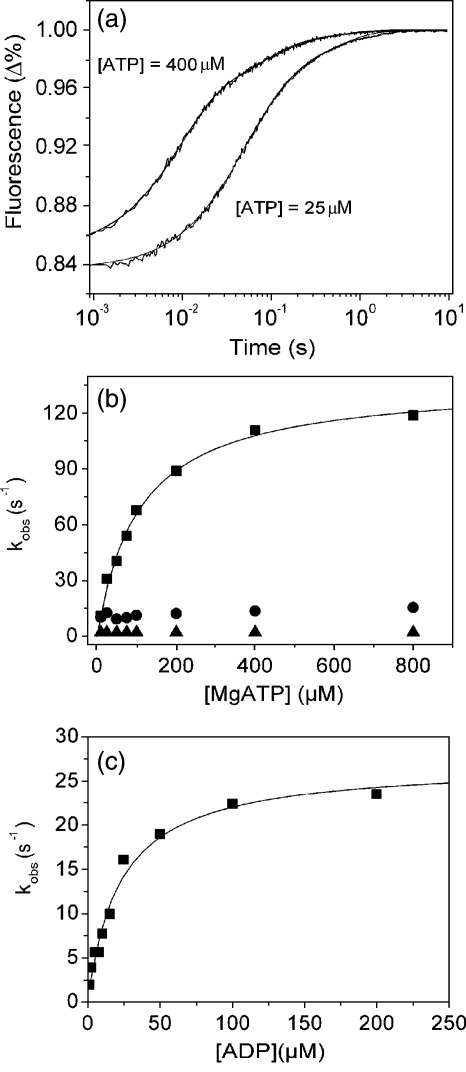
Rate of association of ATP or ADP with ^BM^S1. (a) The protein fluorescence changes observed on mixing 0.5 μM ^BM^S1 with 25 μM ATP or 400 μM ATP at 20 °C. For the reaction with 25 μM ATP the best-fit double exponential is superimposed with *k*_obs_ = 19.6 s^−1^ (Δ*F* +11.8%) and 2.5 s^−1^ (Δ*F* +4%) for the fast and medium/slow phase, respectively. The fluorescence of the reaction with 400 μM ATP could best be fitted to a triple exponential with *k*_obs_ = 111 s^−1^ (Δ*F* +10%), *k*_obs_ = 13.5 s^−1^ ((Δ*F* +3.7%) and *k*_obs_ = 2 s^−1^ (Δ*F* +1%) for the fast, medium and slow phase, respectively. (b) Dependence of *k*_obs_ on ATP concentration. At high ATP-concentrations, *k*_obs_ saturates at 117 s^−1^ for the fast phase (▪), at 18 s^−1^ for the medium phase (•) and 2.0 s^−1^ for the slow phase (▴). At low ATP concentrations the data fit a straight line with slope (*K*_1_*k*_+2_) = 1.5 × 10^6^ M^−1^s^−1^ for the fast phase. (c) Dependence of *k*_obs_ on ADP concentration, with a saturation value for *k*_obs_ = 26 s^−1^. The maximal rate of ADP-binding represents *k*_+6_ +*k*_-6_ (*k*_max,ADP_) = 26 s^−1^ and *K*_7_ = *K*_0.5,ADP_ = 21 μM. The intercept yields *k*_+6_ (=*k*_-D_) = 1.4(±0.5) s^−1^. The second-order constant of ADP binding *k*_-6_/*K*_7_ (=*k*_+D_) is defined by *k*_max,ADP_/*K*_0.5,ADP_ = 1.24 × 10^6^ M^−1^s^−1^, resulting in *K*_D_ (*k*_-D_/*k*_+D_) = 1.2 μM.

MHC-II-1 myosin was purified from bovine masseter muscle fibres (see Materials and Methods). Figure 1 shows the isolated myosin with two light chains and the purified ^BM^S1 with only one light chain bound. The binding of ATP or ADP to myosin S1 is characterised by an increase in intrinsic protein fluorescence associated with nucleotide binding. Figure 2(a) shows the fluorescence change observed on rapidly mixing 25 μM or 400 μM ATP with 0.5 μM ^BM^S1 at 20 °C (concentrations are post mixing). At 25 μM the transient increase in fluorescence (Δ*F*) shown can be satisfactorily described by a two exponential function with *k*_obs_ = 19.6 s^−1^ (Δ*F* +11.8%) and 2.5 s^−1^ (Δ*F* +4%). The fast phase is easily separated from the slower component and at low ATP concentrations (<50 μM) appears linearly dependent on ATP concentration with an apparent second-order rate constant of 0.97 × 10^6^ M^−1^s^−1^. At higher ATP concentrations the *k*_obs_ value saturates and a hyperbolic fit to the data gives a *K*_0.5_ value of 56 μM with a *k*_max_ of 117 s^−1^ (Figure 2(b)). The slow phase only appears as a single process at low ATP concentrations (≤50 μM); at higher ATP there appear to be two components. Separating out these two phases across the full range of ATP concentrations is problematic but at ATP concentrations above 50 μM, the data can all be described by a small slow phase of amplitude 1–1.5% with a *k*_obs_ of 2 s^−1^ and a medium rate phase with *k*_obs_ of 12-15 s^−1^ and an amplitude of 4%. This is illustrated in Figure 2(a) for [ATP] = 400 μM where the transient increase in fluorescence (Δ*F*) shown can be satisfactorily described by a triple exponential function (with *k*_obs_ = 111 s^−1^ (Δ*F* +10%), *k*_obs_ = 13.5 s^−1^ (Δ*F* +3.7%) and *k*_obs_ = 2 s^−1^ (Δ*F* +1%) for the fast, medium and slow phase, respectively). The analysis of the data shown in Figure 2(b) has been fitted assuming a constant slow phase of 2 s^−1^. The medium phase process at 12–15 s^−1^ is then only apparent when the fast component is faster than ∼30 s^−1^ and then appears to be constant. In order to rule out contamination with ADP as the cause of the slower phases, ^BM^S1 was extensively treated with apyrase but this had little effect on the observed transients.

**Scheme 1:**
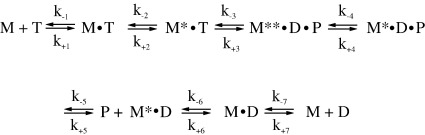
Proposed reaction scheme for the interaction of ^BM^S1 with nucleotides (T, ATP; D, ADP) as described.^43^

The *K*_0.5_ and *k*_max_ of the fast phase can be assigned to *K*_1_ and *k*_+2_ of Scheme 1, respectively, the steps thatcontrol ATP binding as observed for many other myosin isoforms. One of the two slower phases could be due to the ATP hydrolysis step of ATP (*k*_+3_ + *k*_-3_ in Scheme 1) that is known to generate changes in fluorescence for other myosins as described.^20^^,^^21^ In order to determine what is being observed here, quench flow measurements were done in order to establish the rate of ATP hydrolysis.

**Figure 3:**
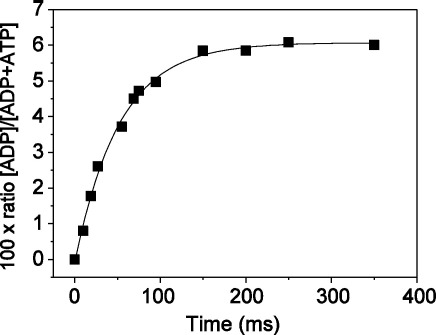
Kinetics of MgATP hydrolysis by ^BM^S1 using rapid quench-flow at 20 °C (100 mM KCl, 20 mM Mops, 5 mM MgCl_2_(pH 7)). 5 μM ^BM^S1 is first mixed with 50 μM MgATP and after variable time quenched with 6.25% TCA The ADP/total nucleotide ratio is plotted as a function of time and can be fitted to a single exponential, resulting in a time constant *t* for the initial burst (*t* = 52 ms), resulting in a *k*_obs_ of 19 s^−1^.

^BM^S1 (5 μM) was mixed with excess ATP (50 μM) and incubated at 20 °C for desired time intervals (up to 350 ms). Figure 3 shows a rapid initial burst of ADP production, which can be described by a single exponential function with *k*_obs_ of 19 s^−1^ and an amplitude of 0.61 ADP/S1. The amount of irreversibly bound ATP to ^BM^S1 at the end of the burst was determined by quenching the reaction into a hexokinase/glucose buffer after 1 s and then adding 6.5% trichloroacetic acid (TCA) after a further 1 s. In controls this treatment hydrolysed any free ATP to ADP. After HPLC analysis of the reaction mixture 0.14 ATP/S1 remained and therefore at the end of the burst phase 0.14 ATP/S1 remains as tightly bound M*.ATP and 0.61 ADP/S1 as M**.ADP.Pi (Scheme 1). Thus the value of *K*_3_ = [M.ADP.Pi]/ [M*.ATP] = 4.3. This is similar to the values reported for fast muscle S1.^22^^,^^23^ The value of the *k*_obs_ can be assigned to *k*_+3_ + *k*_-3_ and at 19 s^−1^ is similar to the medium phase of the stopped-flow fluorescence transient (*k*_obs_ = 15 s^−1^). Thus, as for other myosins the hydrolysis step is accompanied by a fluorescence change. It is worth noting that this is quite a slow process, compared to the hydrolysis rate for fast skS1 from rabbit, which is tenfold faster, but similar to the hydrolysis rate for the slow myosin S1 from rabbit or pig soleus muscle^9^ (see Table 1). The origin of the slowest component of the fluorescence transient remains to be defined. This is small (1–1.5%) and has a constant *k*_obs_ value of 2 s^−1^. This could arise from a small fraction of damaged myosin, from a contaminant myosin or from a fraction of the myosin that must isomerise before ATP can bind to it (see Discussion of ATP binding to actomyosin). The signals are very weak and represent a small fraction of the total making it very difficult to investigate and we have not pursued this further. Arrhenius plots of the maximal observed rates of the fast phase (*k*_+2_) and the medium phase (*k*_+3_ + *k*_-3_) and van 't Hoff plot of *K*_1_ (not shown) show that between 8 °C–20 °C *K*_1_ does not depend on temperature. *k*_+2_ and *k*_+3_ + *k*_-3_ have a marked temperature dependence with similar activation energies of 71 kJ/mol and 75 kJ/mol, respectively. The ratio of the amplitudes for the fast and medium phase remains fairly constant between 8 °C–20 °C (ratio ∼ 4:1).

The rate of ADP-association with ^BM^S1 was also measured. In this case the fluorescence increase was smaller (Δ*F* 3%) than seen for ATP. There was no evidence of a slow component of the reaction and the transient increase in fluorescence could be fitted to a single exponential. At high ADP concentrations *k*_obs_ reaches a maximum value of 26 s^−1^ when fitted to a hyperbola (Figure 2(c)). The ADP-release rate *k*_+6_ (=*k*_-D_) can be determined from the intercept, *k*_+6_ = 1.4(±0.5) s^−1^. The maximal rate of ADP-binding represents *k*_+6_ + *k*_-6_ (*k*_max,ADP_) = 26 s^−1^ and *K*_7_ = *K*_0.5,ADP_ = 21 μM. The second-order constant of ADP binding *k*_-6_/*K*_7_ (or *k*_+D_) can be defined by *k*_max,ADP_/*K*_0.5,ADP_ = 1.24 × 10^6^ M^-1^s^−1^ (see also Table 1) from which a value for the equilibrium dissociation constant can be calculated: *K*_D_ (1/K_6_*K*_7_) = 1.2 μM. Repeating the ADP-binding experiment using the slow myosin S1 isolated from pig soleus muscle, ^PS^S1, showed that this myosin S1 had a slightly tighter affinity for ADP (*K*_D_ = 0.9 μM; see Table 1).

**Figure 4:**
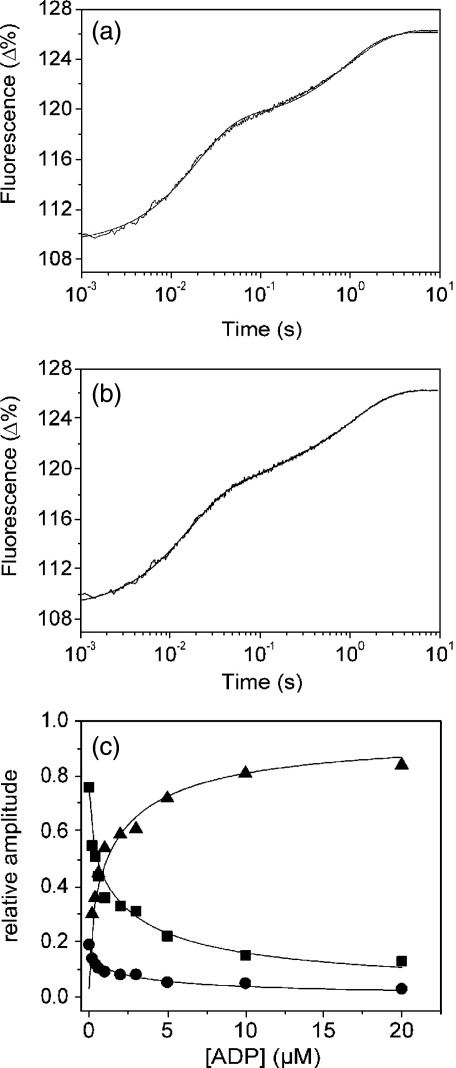
Displacement of ADP from ^BM^S1-ADP by addition of excess ATP. (a) and (b) Protein fluorescence changes observed on mixing 200 μM ATP with 1.0 μM ^BM^S1 and 0.4 μM ADP. Comparison of the sum of two exponentials (a) or three exponentials (b) shows that the data fit best to a sum of three exponentials. The measured values of the *k*_obs_ in (b) were 68 s^−1^ (fast phase, Δ*F* +8.9%), 9.6 s^−1^ (medium phase, Δ*F* +2.2%) and 0.9 s^−1^ (slow phase, Δ*F* +6%), respectively. (c) Dependence of the relative amplitudes of the three exponentials on ADP concentration. The data are fitted to hyperbolae with a *K*_d_ of 2.6 μM (fast phase, ▪), 3.0 μM (medium phase, •) and 3.0 μM (slow phase, ▴).

An alternative method of determining *k*_+6_ (or *k*_-D_) is the displacement of ADP from ^BM^S1 by addition of an excess ATP. Figure 4(a) and (b) shows the reaction observed on adding 100 μM ATP to 0.5 μM ^BM^S1 in the presence of 0.2 μM ADP. The best fit of the fluorescence changes is the sum of three exponentials with *k*_obs_ of 68 s^−1^, 9.6 s^−1^ and 1.1 s^−1^. Two of the observed three phases are due to the binding of ATP to ADP-free ^BM^S1 (68 s^−1^) and the hydrolysis step (9.6 s^−1^), as described above, and the third phase, the binding of ATP, is limited by ADP release from ^BM^S1-ADP (see Figure 4(a) and (b)), with an ADP-off rate of 1.1 s^−1^. Using a triphasic fit the observed rate constants are fairly independent of ADP concentration (average rate constants for the fast, medium and slow phase are 75 s^−1^, 8 s^−1^ and 0.9 s^−1^, respectively) but the measured amplitudes depend on the ADP concentrations. The amplitude of the fast phase (ATP-binding to ^BM^S1) and medium phase (ATP-hydrolysis) decrease and of the slow phase (ADP dissociation) increases as the ADP concentration is increased (Figure 4(c)). The relative ratio of the amplitudes for the fast and medium phase remain constant up to 2 μM ADP (*A*_fast_: *A*_medium_ ∼ 4: 1). At higher ADP concentrations the amplitude of the ATP hydrolysis process, *A*_medium_, becomes very small but the observed rate constant, *k*_obs_, is not affected by [ADP]. In order to be able to separate *A*_fast_ and *A*_medium_ at high ADP concentrations, the relative ratio *A*_fast_/*A*_medium_ was fixed at 4. The ADP dependence of the amplitudes could be described by hyperbolas with independent best-fit dissociation constants of 2.6 μM (fast phase), 3.0 μM (medium phase) and 3.0 μM (slow phase), resulting in an average *K*_D_ = 2.8 μM. This is in reasonable agreement with the value found previously using the ADP binding experiment in Figure 2 (*K*_D_ = 1.2 μM) and we will use the median value of 2.0. A tighter affinity of ADP for the S1 product of bovine cardiac MHC-1 myosin was reported (*K*_D_ = 0.33 μM) when the interaction of ATP and ADP with bovine cardiac S1 was investigated.^11^

### Nucleotide binding to MHC1 myosin S1 in the presence of actin

**Figure 5:**
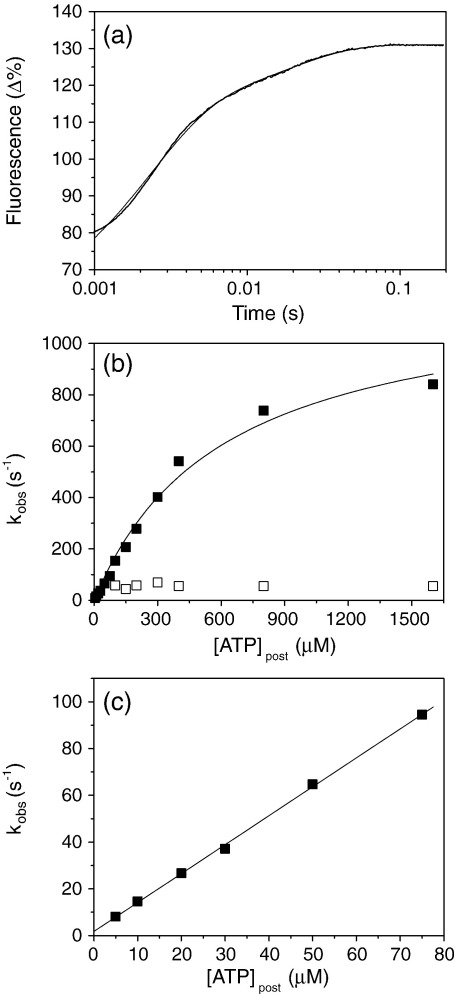
ATP-induced dissociation of pyr-acto–^BM^S1. (a) The pyrene fluorescence changes observed on mixing 0.5 μM ^BM^S1 with 200 μM ATP at 20 °C. The best-fit double exponential is superimposed with *k*_obs_ = 278 s^−1^ (Δ*F* +77%) and 58 s^−1^ (Δ*F* +10%) for the fast and slow phase, respectively. (b) Dependence of *k*_obs_ on [ATP]. At high ATP-concentrations, *k*_obs_ saturates at ∼1100 s^−1^ for the fast phase and remains constant at 56(±13) s^−1^ for the slow phase. (c) At low [ATP] the data of the fast phase fit a straight line with slope (*K*_1_*k*_+2_) = 1.23 × 10^6^ M^−1^s^−1^.

The ATP-induced dissociation of acto–MHC-1 complexes can be followed by monitoring the fluorescence of a pyrene label covalently attached to Cys374 of actin. At ATP concentrations above 100 μM, the ATP-induced dissociation of bovine masseter myosin S1 (^BM^S1) is biphasic, as is illustrated in Figure 5(a) and (b), whereas below 100 μM ATP the fluorescence can be fitted to a single exponential. Figure 5(a) shows the resulting change in fluorescence upon mixing 0.5 μM pyrene-labelled acto–^BM^S1 complex with 200 μM ATP. At this ATP concentration the reaction can be best described by a double exponential with *k*_obs_ = 278 s^−1^ (Δ*F* 77%) and 58 s^−1^ (Δ*F* 10%) for the fast and the slow phase, respectively. The fast phase shows a hyperbolic dependence on [ATP] and saturates at *k*_obs_ = 900–1100 s^−1^ whereas the slow phase remains constant at 56(±13) s^−1^ at [ATP] > 100 μM (Figure 5(b)). The rate constant of the slow phase (*k*_slow_ = 56 s^−1^) is similar to the rate constant of ADP-release (94 s^−1^) and one could argue it is possible that the slow phase is due to contamination with ADP. However, prolonged incubation of ^BM^S1 with apyrase did not remove the slow phase. The presence of another myosin isoform contamination might also cause the biphasic behavior. Bovine masseter fibres are not 100% MHC-1β but contain a small amount (5%) of MHC-1α isoform but this is a fast isoform and is therefore unlikely to cause an additional slow phase.^13^ The presence of non-muscle myosins (either class 2 or others) could produce a slow phase but the amplitude of the slow phase (15%) is far too large to come from such a source. For ^BM^S1, according to scheme II, the *k*_obs_ for the slow phase observed here can be assigned to *k*_+α_ = 56 s^−1^. From the ratio of the amplitudes of the fast and slow phase of ATP-induced acto–^BM^S1 dissociation (77:10) the value of *K*_α_ = 7.7, can be assigned. Since *K*_α_ = *k*_+α_/*k*_-α_ and *k*_+α_ = 56 s^−1^ then *k*_-α_ = 7.3 s^−1^. The temperature-dependence of the ATP-induced dissociation of acto–^BM^S1 was measured between 8 °C −32 °C at a low ATP-concentration (50 μM). From the dependence of *k*_obs_ on temperature the activation energy can be calculated as *E*_a_ = 22 kJmol^−1^ (not shown).

**Figure 6:**
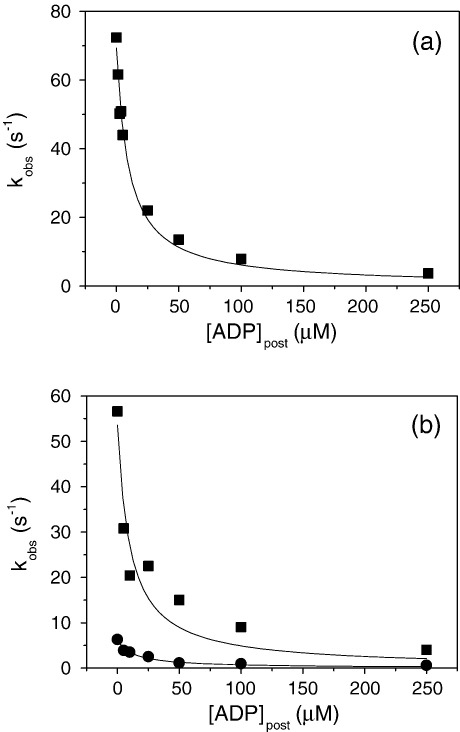
ATP-induced dissociation of pyr-acto–^BM^S1 in the presence of ADP. 0.25 μM phalloidin-stabilized pyrene-labelled actin was incubated with an equimolar amount of ^BM^S1 before mixing with variable concentrations of ADP and 50 μM ATP (a) or 200 μM ATP (b). The data could be fitted against a single exponential (a) or a double exponential (b) and the resulting *k*_obs_ were fitted toa hyperbola, resulting in an apparent affinity (*K*_AD_) forADP (a) *K*_AD_ = 9.6(±1.4) μM (20 °C) and (b) *K*_AD_ = 6.8(±1.7) μM (▪, fast phase) and *K*_AD_ = 6.6(±1.9) μM (•, slow phase).

The effect of ADP on the dissociation rate constant was measured at two different ATP concentrations: [ATP] = 50 μM and [ATP] = 200 μM. At [ATP] = 50 μM, the ATP-induced dissociation of 0.5 μM pyrene-labelled acto–^BM^S1, pre-incubated with 0–250 μM ADP, yielded a fluorescence change of 55% that could be fitted to a single exponential. The resulting *k*_obs_ was plotted against ADP concentration (see Figure 6(a)) and fitting against equation (4) resulted in a *K*_AD_ of 9.6(±1.4) μM. Presuming the on-rate is diffusion- limited (∼10^7^ M^−1^s^−1^) one can calculate the ADP-off-rate (*k*_-AD_ = 96 s^−1^). Reducing the temperature to 12 °C reduced the measured *K*_AD_ to 5 μM and thus also the calculated ADP off-rate (50 s^−1^). At higher ATP concentration (200 μM) the fluorescence change of the ATP-induced dissociation of ^BM^S1-actin, in the absence of ADP, can be fitted to a double exponential and both *k*_obs_ values decreased with increasing ADP concentrations. Plotting the *k*_obs_ values *versus* the ADP-concentration (Figure 6(b)) yielded *K*_AD_ values of 6.8(±1.7) μM (fast phase) and 6.6 μM(±1.9) μM (slow phase), indicating that the *k*_obs_ of both phases are inhibited by ADP.

**Figure 7:**
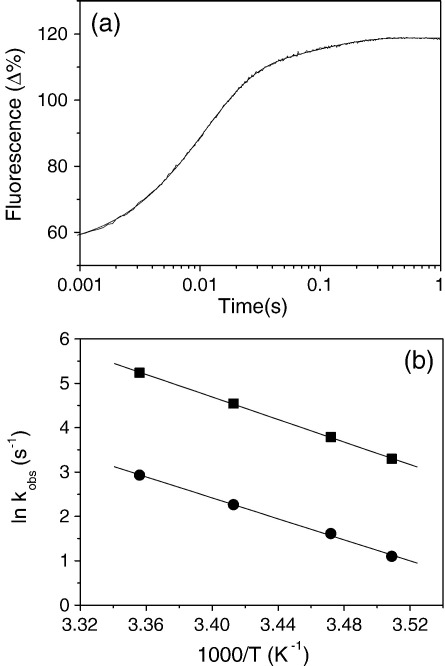
ATP-induced dissociation of ^BM^S1-actin in the presence of ADP. (a) Pyrene fluorescence changes observed on mixing 16 mM MgATP with 1 μM ^BM^S1-actin in the presence of 150 μM ADP at 20 °C. The best-fit double exponential is superimposed with *k*_obs_ = 94 s^−1^ (Δ*F*+55%) and 9.6 s^−1^ (Δ*F* +10%) for the fast and slow phase, respectively. (b) Temperature dependence of ATP-induced dissociation of ^BM^S1-actin in the presence of ADP with *k*_obs_ (fast (▪) and slow (•) phase) as a function of temperature. From the slope the activation energy can be calculated. *E*_a_ = 106 kJmol^−1^ and 98 kJmol^−1^ for the fast and the slow phase, respectively.

**Scheme 2:**
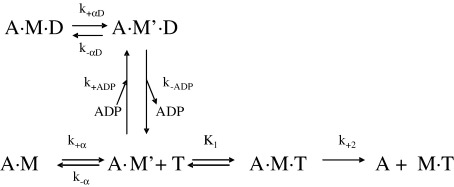
Proposed reaction scheme for the interaction of ^BM^S1 with nucleotides (T, ATP; D, ADP) in the presence of actin as proposed for myosin I (A represents actin and M is myosin S1).^44^

The temperature dependences of these two phases are measured at very high ATP concentrations ([MgATP] = 16 mM) in order to define the rate of ADP release. The ATP-induced dissociation of the acto–^BM^S1 complex in the presence of saturating ADP concentrations (150 μM) is biphasic with a fast *k*_obs_ = 94 s^−1^ (Δ*F* 55%) and a slow *k*_obs_ = 9.6 s^−1^ (Δ*F* 10%) at 20 °C (see Figure 7(a)). According to the model depicted in Scheme 2, these two processes are due to the presence of two actomyosin complexes with ADP bound in solution, A·M'·D and A·M·D. The fast phase measured here (94 s^−1^) is due to the A·M'·D complex and corresponds well with the calculated ADP off-rate (96 s^−1^) and, according to Scheme 2 represents *k*_-ADP_. The other complex, A·M·D, is responsible for the slow phase observed here and, according to Scheme 2, represents the rate-limiting step of ADP-release (*k*_+αD_ = 9.6 s^−1^). Between 12 °C–25 °C the temperature dependences of these observed fast and slow rates are almost identical (see Figure 6(b)) with similar activation energy values *E*_a_ = 106 kJ (fast phase) and 98 kJ (slow phase). The ratio of amplitudes of the two phases, *A*_fast_:*A*_slow_ had a small dependence on the temperature with *A*_f_:*A*_s_ = 6.3 at 12 °C and 5.1 at 25 °C.

**Figure 8:**
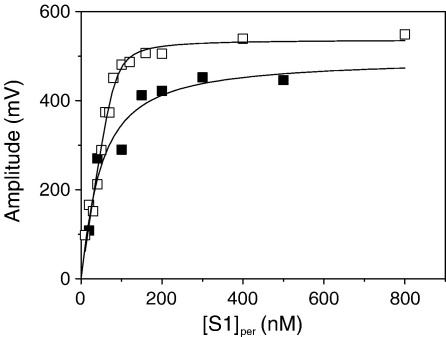
Titration of actin with ^BM^S1. 30 nM phalloidin-stabilised pyrene-labelled actin was incubated with various amounts of ^BM^S1 before mixing with 500 μM ATP in the presence of 500 μM ADP (▪) or with 20 μM ATP without ADP (□). In both cases the reaction was well described by a single exponential and the amplitude increased with increasing [^BM^S1]. The best fit to the quadratic equation describing the binding isotherm (see Materials and Methods) gave a *K*_d_ of 7 nM and 37 nM in the absence and presence of ADP, respectively (concentrations used here are before mixing).

The small thermodynamic coupling (*K*_AD_/*K*_D_) between actin and ADP binding to ^BM^S1 (*K*_AD_/*K*_D_ = 4.8) can be validated by measuring the affinity of S1 for actin in the presence and absence of ADP (*K*_DA_/*K*_A_). Phalloidin-stabilised pyr-actin (15 nM) incubated with various concentrations of ^BM^S1 were mixed with ATP (10 μM), and the amplitude of the dissociation reaction was used to estimate the fraction of actin bound to ^BM^S1. Without ADP present, *k*_obs_ remained constant at all S1 concentrations (*k*_obs_ = 19 s^−1^) and the amplitude increased hyperbolically with [^BM^S1]. In the presence of 250 μM ADP, the value for *k*_obs_ was reduced (15 s^−1^) and the amplitudes increased hyperbolically with [S1] (see Figure 8). In the absence of ADP the best fit to the titration gave a *K*_A_ ∼ 4–7 nM, whereas in the presence of ADP the best fit yielded a *K*_DA_ of 37 nM. Thus, ADP reduced the affinity of S1 for actin by about fivefold (*K*_DA_/*K*_A_ = 37/7 = 5.2), which is similar to the reduction in ADP affinity for S1 caused by actin (*K*_AD_/*K*_D_ = 4.8).

Using MHC-II-1 myosin S1 from pig soleus muscle (^PS^S1) very similar results were obtained and these are summarised in Tables 1 and 2. The ATP-induced dissociation of acto–^PS^S1 was biphasic at high ATP with a fast phase (*k*_max_ = 800–900 s^−1^) and a slow phase (*k*_max_ = 67 s^−1^), and a relative ratio *A*_fast_:*A*_slow_ = 4.8 (data not shown). This yields a value for *K*_α_ = 4.8, and together with the measured value for *k*_+α_ = 67 s^−1^ gives *k*_-α_ = 14 s^−1^ (Table 2). In the presence of ADP (0–200 μM) the ATP-induced dissociation of acto–^PS^S1 also gave fluorescence transients that fitted a single exponential at low ATP concentrations (<100 μM) and a double exponential at high ATP concentrations (data not shown) and a *K*_AD_ of 15 μM. At very high ATP concentrations (4 mM MgATP) the ATP-induced dissociation of the acto–^PS^S1 complex in the presence of saturating ADP concentrations (50 μM) is also biphasic with a fast rate *k*_obs,f_ = 48 s^−1^ and a slow phase *k*_obs,s_ = 6.5 s^−1^ at 20 °C. The ratio of the two amplitudes *A*_fast_:*A*_slow_ is 6.5 (20 °C) and represents *K*_αD_ (see Table 2).

## Discussion

This paper set out to reexamine and extend the kinetic analysis of the bovine MHC-II-1 isoform of myosin found in slow skeletal muscle and the ventricle of cardiac muscle. Bovine masseter muscle fibres were successfully used to isolate the S1 construct (^BM^S1) of bovine MHC-II-1 myosin. Comparison of the kinetic parameters of ^BM^S1 and the S1 construct isolated from the left ventricle of bovine cardiac tissue (^BC^S1),^10^^,^^11^ which is 100% MHC-II-1 and has the same light chain content, shows that theresults agree reasonably well with each other (Tables 1 and 2). The two parameters that deviate significantly between ^BM^S1 and ^BC^S1 are discussed below.

The ATP hydrolysis rate constant reported here is almost tenfold slower than that reported.^12^ The previously reported rate constant was based on the assumption that when ATP binds to S1, the maximum rate of the fluorescence change represents *k*_+3_ + *k*_-3_ (Scheme 1), in analogy with measurements for fast myosin S1.^20^^,^^21^ However, the quench flow data reported here show that *k*_+3_ + *k*_*-*3_ defines the medium phase observed in the stopped flow measurements and the maximum observed rate constant of the medium phase is *k*_+3_ + *k*_-3_ = 19 s^−1^, whereas the fast phase represents *k*_+2_. The ATP hydrolysis step of ^BM^S1 is much slower than that of fast skeletal S1 from rabbit (*k*_+3_ + *k*_-3_ = 131 s^−1^) but it is similar to the slow skeletal myosin II, MHC-II-1 from rabbit soleus (22 s^−1^)^9^ and pig MHC-II-1 (24.5 s^−1^).^24^ It may therefore be a general feature of slow myosin II isoforms. The role of this reduced ATP hydrolysis rate for MHC-II-1 could be related tothe slow ADP-release rates also observed for MHC-II-1. A slow ADP-release rate increases the time myosin is bound to actin (i.e. enhances the duty ratio). MHC-II-1 can compensate for this by a slow hydrolysis step thereby maintaining the low duty ratio typical of striated muscle myosins.

The affinity of ADP for ^BM^S1 in the absence of actin (*K*_D_ = 2 μM) is weaker than the value reported for ^BC^S1 (*K*_D_ = 0.33 μM) when the interaction of ATP and ADP with ^BC^S1 was investigated.^11^ Due to aggregation problems in the ^BC^S1 work, the interaction with ATP was reinvestigated a year later^12^ but as far as we are aware, the interaction with ADP has never been re-examined and this may therefore account for the discrepancy between the *K*_D_ values. Table 1 shows that the k_+6_ values measured for ^BM^S1 and ^BC^S1 differ two- to threefold (*k*_+6_ = 0.9–1.4 s^−1^ and 0.5 s^−1^, respectively) while the values for *k-*_6_/*K*_7_ are fairly similar (1.24 × 10^6^ M^−1^s^−1^ and 1.4 × 10^6^ M^−1^s^−1^). The revised *K*_D_ value for bovine MHC-II-1 S1 has implications for the thermodynamic coupling constant between the affinity of myosin for ADP and actin, *K*_AD_/*K*_D_ (see below).

Comparison of fast and slow muscle myosin II isoforms shows that in the absence of actin, most kinetic parameters are quite similar (Table 1). The major exception is the ATP-hydrolysis rate (*k*_+3_ + *k*_-3_), which is about tenfold slower for the slow MHC-II-1 S1 isoforms compared to rabbit psoas S1. The ADP dissociation rate constant (*k*_+6_) is very similar for all slow isoforms (*k*_+6_ 0.5–0.9 s^−1^) and about twofold slower than the ADP dissociation rate constant of fast skeletal S1 (*k*_+6_ = 1.4 s^−1^). The ADP association rate constant (*k*_-6_/*K*_7_) is similar for all slow isoforms. The different estimates of ADP binding and release result in a surprisingly wide range of values for the ADP-affinity for S1 isoforms listed from 0.4 μM (rabbit soleus) to 2 μM (bovine masseter). However, these *K*_D_ values are well defined by both the ratio of rate constants (Figure 2) and the amplitude titration data (Figure 4), suggesting that this parameter does vary across the series of isoforms.

The data in the presence of actin (Table 2) indicate that the affinity of S1 for actin in the presence (*K*_DA_) and absence (*K*_A_) of ADP shows a sursprisingly large variation within the group of slow isoforms with a tenfold difference in *K*_A_ value of bovine masseter andrabbit soleus and an even larger difference in *K*_DA_ value. However, despite these differences in individual equilibrium constants, the slow isoforms show a similar range of values for their thermodynamical coupling constants (*K*_AD_/*K*_D_ and *K*_DA_/*K*_A_). The slow isoforms show ratios of 3–20, all lower than the value for fast rabbit isoforms (30–60) and at the lower end similar to smooth muscle myosin (4–7)^25^^,^^26^ and non-muscle isoforms (∼1–5).

Table 2 shows the parameters that control the ATP induced dissociation of acto–S1 vary relatively little for the fast and slow muscle isoforms listed (*K*_1_*k*_+2_' and *k*_+2_' vary less than twofold). The major difference is that all of the slow isoforms show a two-phase ATP-induced dissociation of acto–S1 whereas the fast muscle protein shows a single phase. Siemankowski & White^10^ stated that the ATP-induced dissociation of acto^BC^S1 (in the presence or absence of ADP) could not be fitted unambiguously to a single exponential, suggesting that it too has two components. Additional indications of two phases comes from data measured for other slow MHC-II-1 isoforms of the chicken, from slow skeletal, cardiac, and smooth muscle^27^ which all show the presence of an additional small slow phase. Thus, a biphasic reaction for ATP-induced dissociation of acto–S1 appears to be common for slow muscle myosin isoforms. ATP-induced biphasic dissociation of acto–S1 has also been reported for a some class I mammalian myosins and non-muscle myosin II.^26^ The biphasic transient has been interpreted in terms of scheme II in which a fraction of the acto–S1 must isomerise before nucleotide can bind.

The work presented here also confirms the complex nature of the ADP release from acto–S1 reported for soleus S1. It is not simply that ADP release is much slower for this group of myosins than for the fast muscle myosin but there is clear evidence for several A.M–ADP complexes. We show here evidence for two complexes with ADP with an equilibrium constant between them (*K*_αD_) of 5.3 and rate constants of ADP release of 94 (*k*_-ADP_) and 9.6 (*k*_+αD_) s^−1^. Similar behavior is seen with the pig soleus (Table 2) and rabbit soleus (Table 3) but with variation across the three slow isoforms particularly in the value of *K*_αD_. The data with rabbit soleus provided evidence for a third AMD complex and by analogy we might expect a rapid collision complex between ADP and acto–S1 not characterised here.

The value of the ADP release rate constant (*k*_-ADP_) has been assigned to the event during the actin-attached part of the ATPase cycle, which limits the maximum shortening velocity (*V*_0_) of a muscle fibre. The velocities of contraction are best characterised at 12 °C (0.17 M ionic strength) and the published values of *V*_0_ for the three slow muscle isoforms are listed in Table 3 along with the values of the ADP release constant at 12 °C. The maximum unloaded shortening velocity of muscle fibres (*V*_0_) of bovine masseter is 0.27 μm s^−1^ at 12 °C.^13^ From this, one can estimate the minimum value of the rate constant that limits the detachment of the cross-bridge (*k*_min_) using the relationship:

$$k_{\text{min}}=\frac{V_{0}}{d}$$

with *V*_0_ = maximum sliding velocity (nm s^−1^) and *d* = working stroke (nm).^8^ Depending on the size of the working stroke, *k*_min_ is 54 s^−1^ (*d* = 5 nm) or 27 s^−1^ (*d* = 10 nm) for bovine masseter MHC-II-1. From Table 3 one can see that at 12 °C *k*_-ADP_ is 27 s^−1^, which is close to the calculated *k*_min_ value. The data from the pig shows a similar correlation between *k*_min_ and *k*_-ADP_, as does the previously published data from rabbit.^9^ For all three MHC-II-1 isoforms the measured values of *k*_+αD_ are much slower than the calculated *k*_min_, indicating that this isomerisation step cannot be part of the main pathway of the unloaded cross-bridge cycle but represents a branched pathway that could become significant if the motor is contracting under loaded conditions. The velocity measurements are, by definition, made under zero load and the isomerisations controlling the ADP release steps are events which are potentially sensitive to the load on the actomyosin cross-bridge.

The evidence that ADP release is load-dependent has been discussed in general terms by Nyitrai & Geeves^28^ and specifically for the rabbit MHC-II-1 isoform by Iorga *et al*.^9^ The data for bovine and porcine isoforms are shown here to be similar to the rabbit isoform and a similar interpretation is possible. Briefly, there is substantial evidence from single-molecule mechanical measurements^29–32^ and electron micrographs of S1 decorated actin filaments^17–19^ that, for a range of non-muscle myosins and the smooth-muscle myosin there is a load-dependent structural change after the major force generating event. The force-generating event is associated with phosphate release and the second structural change is probably associated with ADP release. More recent studies with improved laser traps^33^ and X-ray scattering from S1-decorated, orientated actin filaments^16^ have extended these observations to striated muscle myosins. The laser trap observed a ∼1 nm movement of the lever arm in the second step for the rat MHC-II-1 isoform and the rate constant we expect for the event limiting ADP release is compatible with the lifetimes measured in the laser trap. The X-ray data report a similar sized movement of the lever arm on binding/release of ADP for the rabbit slow muscle S1. Together, the structural mechanical and biochemical kinetic studies support the hypothesis of a load dependent isomerisation of the actomyosin complex required to allow release of ADP.

The results presented here on the protein isomerisations that limit ADP release from the actomyosin of slow muscle may provide some insight into why, despite a tight affinity for ADP, slow muscles are more resistant to fatigue than fast muscles, or at least the part of fatigue associated with ADP build up. Fatigue is classically defined from studies of the effect of prolonged stimulation on the isometric tension of intact muscle fibres. The complete explanation of the loss of tension remains to be defined but any build up of ADP is likely to result in the enhancement of the lifetime of strongly bound A.M.–ADP states as ADP competes with ATP induced detachment of the cross-bridge. The increased lifetime will increase steady-state tension as is seen on addition of ADP to skinned muscle fibres.^34–36^ Thus, ADP build up may compensate for other effects of fatigue which tend to reduce tension and the compensatory effect will be greater in slow than in fast muscle fibres.

The effect of ADP on velocity is different; the evidence is for a slowing of maximal shortening velocity in skinned muscle fibres.^34^^,^^37^ In a fast muscle the velocity is limited by the rate of ATP-induced dissociation of actin from A.M., and ADP competes with ATP for binding to the nucleotide pocket.^38^ The net rate constant for actin detachment is defined by:

$$k_{\text{detach}}=K_{1}k_{+2}[\text{ATP}]/(1+K_{1}[\text{ATP}]+[\text{ADP}]/K_{\text{AD}})$$

Thus, at mM ATP concentrations, ADP of the order of the *K*_AD_ (∼100 μM) can produce a significant inhibition of the detachment rate constant. For slow muscle the detachment of myosin from actin is limited not by the ATP induced reaction but by the slower isomerisation preceding ADP release, controlled by *k*_-ADP_ (Table 3 gives this as 27 s^−1^ for masseter at 12 °C). The following ATP-induced dissociation of AM remains much faster than this (∼1000 s^−1^ at mM ATP concentrations, in fact the *K*_1_, and *k*_+2_ values are similar for fast and slow myosin isoforms; see Table 2) and the velocity will only be slowed once the ADP competition effect reduces *k*_detach_ to <100 s^−1^ (equation (7)). This requires the ADP concentration to be many times the *K*_AD_ value or for the ATP concentration to fall to ∼100 μM.

In summary we have shown that the MHC-II-1 isoforms isolated from slow skeletal muscles from the cow and the pig are similar in general properties to MHC-II-1 isolated from bovine cardiac tissue and to the MHC-II-1 of the rabbit and these are quite distinct from the MHC-II-2 isoforms. Of significance is the slow ATP-hydrolysis rate, the low thermodynamic coupling between ADP and actin binding, the slow rates of ADP release (which are of the correct size to limit the maximum shortening velocity) and multiple actomyosin ADP complexes. Nucleotide access to and release from the binding site requires relatively slow isomerisations and these may be load-dependent. These properties are distinct from the fast muscle MHC-II-2 isoforms and similar to smooth and some non-muscle myosin isoforms, e.g. mammalian myosins 1b and 1c, and non-muscle myosin II. However, unlike the non-muscle isoforms, the MHC-II-1 isoforms have a low duty ratio and can produce relatively rapid movement in motility assays or in muscle fibres. These parameters suggest that slow muscle myosin isoforms may form a distinct functional group from the fast muscle isoforms and the slower group which we have called the strain-sensors; non-muscle myosins particularly a sub-set of class I myosins (myo1b and 1c) that appear to operate less as transport motors than as load-bearing and strain-sensing molecules. Slow muscle myosin, like fast muscle myosin, is required for movement but also has an additional role in efficient load bearing in postural muscles. Thus, the molecular properties of the MHC-II-1 motor are consistent with its physiological role.

## Materials and Methods

### Proteins

MHC-II-1 myosin was purified from bovine masseter muscle fibres that had been frozen in liquid nitrogen prior to storage at −80 °C. Purification was based on the method described.^39^ Briefly, the tissue (40 g) was homogenized in ice-cold Guba-Straub buffer (pH 6.6) and the suspension was stirred for 30 min. After centrifugation, the supernatant was diluted tenfold with water. The precipitated protein was collected by centrifugation. The pellet was taken up in 3M KCl and ice-cold water was then added to precipitate the myosin and the protein was collected by centrifugation. This procedure was repeated three times and the pellet was then taken up in a small amount of 3M KCl and then adjusted to 125 mM KCl, 10 mM KPi, 2 mM EDTA, 4 mM dithiothreitol (DTT, final concentrations) for digestion. Subfragment 1 (^BM^S1) was prepared by digesting myosin using chymotrypsin using the method described.^40^ The soluble ^BM^S1 protein was separated from the non-digested myosin and the insoluble myosin tails by centrifugation and purified over a Q-Sepharose column in 50 mM imidazole (pH 7) with a linear gradient from 0–500 mM KCl. The fractions containing ^BM^S1 were pooled and dialysed into 10 mM KP_i_ buffer (pH 7.5) and typical yields were around 140 mg of protein. Figure 1 shows the isolated myosin with two light chains and the purified ^BM^S1 with only one light chain bound. The gel suggests only one isoform of each light chain and the molecular mass of the essential light chain is consistent with this being the MLC-1b/v isoform expected in slow skeletal and ventricle muscle. For myosin S1, isolated from pig soleus muscle, a similar procedure was followed to isolate the protein and from 58 *g* of tissue about 70 mg of pig soleus S1 (^PS^S1) was obtained. After dialysis, ^BM^S1 and ^PS^S1 were lyophilized in the presence of 1% (w/v) sucrose and stored at −20 °C. Prior to use, ^BM^S1 or ^PS^S1 was dialyzed into 20 mM Mops, 30 mM KCl, 5 mM MgCl_2_, 1 mM sodium azide and remained stable in solution for several days. Rabbit skeletal muscle actin was prepared according to methods described^41^ and, when necessary, labelled with pyrene at Cys374.^42^

### ATPase assay

ATP-ase activity was measured using an NADH-coupled assay at 20 °C in buffer containing 20 mM Mops (pH 7), 5 mM MgCl_2_, 30 mM KCl, 1 mM ATP, 40 units/ml of lactate dehydrogenase, 200 units/ml of pyruvate kinase, 1 mM phosphoenolpyruvate, and 200 μM NADH. Changes in *A*_340_ (ε = 6220 M^−1^cm^−1^) were followed using a UV-VIS spectrophotometer. The basal Mg^2+^-ATPase activity of bovine masseter myosin S1 (0.012 s^−1^) agrees well with the value reported (0.017 s^−1^) for bovine cardiac myosin S1 isolated from bovine cardiac left ventricle.^12^ In the presence of 50 μM actin the Mg^2+^-ATPase activity of ^BM^S1 increased more than tenfold (0.182 s^−1^) and the ATP-ase rate was linearly related to the actin concentration over the range of 0–50 μM. (Not shown).

### Transient kinetics

All kinetic experiments were performed at 20 °C in 20 mM Mops, 100 mM KCl, 5 mM MgCl_2_ and 1 mM azide (pH 7), unless indicated otherwise. All measurements were done with a High-Tech Scientific SF-61 DX2 stopped-flow system. Pyrene actin fluorescence was excited at 365 nm and emission was detected after passing through a KV389 nm cut-off filter (Schott, Mainz, Germany). Tryptophan fluorescence was excited at 295 nm and observed through a WG320 filter. The stated concentrations of reactants are those after mixing in the stopped-flow observation cell (“post”) unless indicated otherwise. Stopped-flow data were analysed using the software provided by Hi-Tech (KinetAsyst) and the Origin software (Microcal).

Without actin present, the kinetics of S1 (either ^BM^S1 or ^PS^S1; M) with nucleotide (T,ATP; D,ADP) were interpreted based on the seven-step model described by Bagshaw *etal*.,^43^ where *k*_+i_ and *k*_-i_ are the forward and reverse rate constants and *K*_i_ (*k*_+i_/*k*_-i_) represents the equilibrium constant of the reaction (Scheme 1).

In the presence of actin the kinetics of the interaction ofS1 with nucleotide were based on the model proposed for myo1b^26^^,^^44^ and is depicted in Scheme 2. In this model, AM exists in two conformations in the absence of nucleotide (A.M and A.M', equilibrium defined by *K*_α_ = *k*_+α_/*k*_-α_) and in the presence of ADP (A.M.D and A.M'.D, equilibrium defined by *K*_αD_ = *k*_+αD_/ *k*_-αD_). In the AM' state, the nucleotide can exchange with the solvent (“open” conformation) but not in the AM state (“closed” conformation). Conversion of M to M' requires opening of the nucleotide binding pocket. The data presented here and summarised in Tables 1 and 2 allow assignment of all the rate and equilibrium constants of Scheme 2. The assignment of the constants to the data is summarized here.

In the absence of ADP, high ATP concentrations induce the dissociation of actin from S1 in two phases. The fast phase represents dissociation from A.M' with:

(1) $$k_{\text{obs},\text{fast}}=K_{1}k_{+2}[\text{ATP}]/(1+K_{1}[\text{ATP}])$$

The slow phase is limited by the isomerisation from A.M to A.M' with the maximum value of *k*_obs,slow_ = *k*_+α_. The ratio of the amplitudes of the fast and the slow phase of the ATP-induced dissociation defines the equilibrium constant between the two conformations in solution and since *K*_α_ = *k*_+α_/*k*_-α_, *k*_-α_ can be determined:

(2) $$A_{\text{fast}}/A_{\text{slow}}=[A.M\prime ]/[A.M]=K_{\alpha }$$

For fast myosin S1, such as ^sk^S1 and ^pso^S1, *K*_α_ >> 1 and only a single phase is observed.

For slow myosin S1, in the presence of ADP, the overall dissociation constant of ADP for the actin-S1 complex (*K*_AD_) is defined by equation (3):

(3) $$K_{\text{AD}}=K_{\text{ADP}}\cdot K_{\alpha D}/(1+K_{\alpha D})$$

where *K*_ADP_ is the dissociation constant for ADP from A.M' and *K*_αD_ the equilibrium constant between AM'D and AMD. If the binding of ADP is a rapid equilibrium event, controlled by *K*_ADP_, ADP and ATP compete effectively for binding to AM and the observed rate constant *k*_obs_ is defined by equation (4):

(4) $$k_{\text{obs}}=k_{0}/(1+[\text{ADP}]/K_{\text{AD}})$$

where *k*_0_ is the observed rate constant of ATP-induced actin–S1 dissociation in the absence of ADP and defined by equation (1). To titrate both A.M complexes to A.M.D, actin–S1 was pre-equilibrated with a range of ADP concentrations before adding excess ATP. ATP-induced dissociation of actin from A.M.D occurs *via* the intermediate A.M'.D and A.M' and is limited by *k*_+αD_. If *k*_+α_ and *k*_+αD_ differ by more than a factor of 3, then both fast and slow amplitudes seen at zero ADP are reduced as ADP is increased and replaced by a new phase with *k*_obs_ = *k*_+αD_ (this is the case for ^BM^S1). The effect of ADP on the amplitude of the fast phase of ATP-induced dissociation of actin-S1 was analysed according to:

(5) $${\text{Amp}}_{\text{fast}}={\text{Amp}}_{0}/(1+[\text{ADP}]/K_{\text{AD}})+C$$

where Amp_fast_ represents the observed amplitude of the fast phase of the reaction, Amp_0_ is the amplitude at zero ADP concentration, [ADP] is the concentration of ADP before mixing, *K*_AD_ is the dissociation equilibrium constant of ADP for actin–S1, and *C* is the end point. The end point of the reaction defined by *C* was defined by the fluorescence at infinite high ADP concentrations. Amplitude is calculated as a percentage of the end point of the reaction, according to:

(6) $$\text{Amplitude}=(\Delta F/F_{t}^{\infty })\times 100$$

Where amplitude, expressed as %, equals the change in fluorescence observed during the reaction (Δ*F*) divided by the fluorescence at the end point of the reaction (*F*_t_^∞^), multiplied by 100.

### Quenched-flow experiments

The quenched-flow experiments were carried out with a Hi-Tech RQF-63 using a buffer containing 100 mM KCl, 5 mM MgCl_2_, 20 mM Mops (pH 7.0). ^BM^S1 (5 μM) was mixed with excess ATP (50 μM) and incubated at 20 °C for desired time intervals (up to 350 ms). The reaction was quenched by the addition of an equal volume of 6.25% (w/v) trichloroacetic acid (TCA). After neutralisation with NaOH and a clarification spin at 3000***g*** for 5 min, the samples were diluted 1:10 with the fast protein liquid chromatography running buffer containing 125 mM KP_i_ (pH 5.5). The separation of ADP and ATP was carried out on a fast protein liquid chromatography (FPLC) system (Amersham Biosciences) using a Hypersil ODS (3 μm) column and an isocratic flow. Integration of the peak areas provided the ratio of ADP and ATP at each time point. The ratio of the ADP to the total nucleotide concentration at each given time point was then used to calculate the hydrolysis rate of the myosin. The amount of irreversibly bound ATP to ^BM^S1 at the end of the burst was determined by quenching the reaction into a 100 mM KCl, 20 mM Mops, 5 mM MgATP buffer containing hexokinase (2 μg/μl) and glucose (1 mM) after 1 s and then adding 6.5% TCA after a further 1 s. In controls this treatment hydrolysed any free ATP to ADP. HPLC analysis of the reaction mixture gives information about the amount of ATP bound to S1 at the end of the burst phase.

## Figures and Tables

**Table 1:** Comparison of the rate and equilibrium constants for the interaction of S1 myosins with nucleotides at 20 °C and 100 mM KCl (pH 7.0)

Parameter	S1(bovine)^a^ masseter	S1 (rabbit)^b^ fast skeletal	S1 (bovine)^c^^,^^d^ cardiac	S1 (rabbit)^e^ soleus	S1 (pig)^a^ soleus
*K*_1_*k*_+2_ (10^6^ M^−1^s^−1^)	0.97	1.9	0.81^d^	1.57	–
*k*_+2_ (s^−1^)	117	>1000^j^	138^i^	191	–
*k*_+3_+*k*_-3_ (s^−1^)	18	131	12/138^i^	21.6	24^h^.5^h^
*k*_+6_ (*k*_-D_) (s^−1^)	0.9^f^–1.4^g^	1.4	0.5	0.61	0.84
*k*_-6_/*K*_7_ (*k*_+D_) (10^6^ M^−1^s^−1^)	1.24^g^	1.5	1.4	1.5	–
*K*_D_ (μM)	1.2^g^–2.8^f^	2	0.33	0.41	0.5–1.4

a This study.

b From Ritchie *et al*.^45^

c From Flamig & Cusanovich.^11^

d From Smith & Cusanovich.^12^

e From Iorga *et al*.^9^

f From ADP-displacement data (Figure 4).

g From ADP + S1 association experiment (Figure 2(c)).

h Pig cardiac S1.^24^

i The observed rate of 138 s^−1^, originally assigned to *k*_+3_ +*k*_-3_, actually represents *k*_+2_ (see Discussion).

j From Miller & Greeves.^20^

**Table 2:** Comparison of rate and equilibrium dissociation constants for the head fragments of different myosins in the presence of actin at 20 °C and 100 mM KCl (pH 7.0) unless indicated otherwise

Parameter	S1 (bovine)^a^ masseter	S1 (rabbit)^b^ skeletal	S1 (bovine)^c^ cardiac	S1(rabbit)^e^ soleus	S1 (pig)^a^ soleus
*K*_1_*k*_+2_' (10^6^ M^−1^s^−1^)	1.24	2.4	2.0	1.72	1.55
*k*_+2_'(s^−1^)	1220	902^f^	>500	1019	730^h^
*k*_+α_ (s^−1^)	56	–	–	45	67^h^
*K*_α_	7.7	–	–	6.2	4.8^h^
*K*_D_ (μM)	2	2	0.33^d^	0.41	0.5–1.4
*K*_AD_ (μM)	9.6	120	6.7	11.3	15
*k*_-ADP_ (s^−1^)	94	–	65	58	48
K_αD_	5.3	–	–	0.26^g^	6.5
*k*_+αD_ (s^−1^)	9.6	–	–	15^g^	8
*K*_A_ (nM)	7	33	6.3	74	–
*K*_DA_ (nM)	37	1000	100	1400	–
*K*_AD_/*K*_D_	4.8	30–60	15–20	21	11–30
*K*_DA_/*K*_A_	5.3	30	16	19	–

a This study.

b From Ritchie *et al.*^45^

c From Siemankowski & White.^10^

d From Flamig & Cusanovich^11^ at 15 °C.

e Rabbit soleus S1 in 30 mM KCl, except *K*_D_.^9^

f From Iorga *et al.*^9^ at 30 mM KCl. Rabbit psoas S1 *k*_+2_ was calculated using the reported value at 12 °C and the temperature dependence of the value over the range 5 °C –15 °C.

g At 10 °C.

h At 12 °C.

**Table 3:** The measured and calculated kinetic parameters for three MHC-II-I isoforms at 12 °C, 100 mM KCl (pH 7)

MHC1	*V*_0_ (μm s^−1^ 0.5 sm^−1^	*k*_min,10_ (s^−1^)	*k*_min,5_ (s^−1^)	*k*_-ADP_ (s^−1^)	*k*_+αD_ (s^−1^)	*k*_-αD_ (s^−1^)	*k*_+α_ (s^−1^)	*k*_-α_ (s^−1^)	*K*_α_	*K*_αD_	*K*_AD_ (μM)	*K*_ADP_ (μM)
Cow	0.27^a^	27	54	27	3.5	0.55	31	5.3	5.8	6.3	4.9	5.7
Pig	0.17^b^	17	34	36	5.7	0.79	70	14.6	4.8	7.2	15	17.1
Rabbit^c^	0.67^d^	67	134	63	15	7.9	40	9.5	4.2	1.25	6	3.3

a From Tonilo *et al*.^13^

b From Tonilo *et al*.^15^

c Kinetic data from Iorga *et al*.^9^

d From Pellegrino *et al.*^46^

## References

[1] De La Cruz E.M., Ostap E.M. (2004). Relating biochemistry and function in the myosin superfamily. Curr. Opin. Cell Biol..

[2] Geeves M.A., Holmes K.C. (2005). The molecular mechanism of muscle contraction. Adv. Protein Chem..

[3] Reggiani C., Bottinelli R., Stienen G.J. (2000). Sarcomeric myosin isoforms: fine tuning of a molecular motor. News Physiol. Sci..

[4] Timson D.J. (2003). Fine tuning the myosin motor: the role of the essential light chain in striated muscle myosin. Biochimie.

[5] Schiaffino S., Reggiani C. (1996). Molecular diversity of myofibrillar proteins: gene regulation and functional significance. Physiol. Rev..

[bib6] Lowey S., Waller G.S., Trybus K.M. (1993). Skeletal muscle myosin light chains are essential for physiological speeds of shortening. Nature.

[7] Sherwood J.J., Waller G.S., Warshaw D.M., Lowey S. (2004). A point mutation in the regulatory light chain reduces the step size of skeletal muscle myosin. Proc. Natl. Acad. Sci. USA.

[8] Siemankowski R.F., Wiseman M.O., White H.D. (1985). ADP dissociation from actomyosin subfragment 1 is sufficiently slow to limit the unloaded shortening velocity in vertebrate muscle. Proc. Natl Acad. Sci. USA.

[9] Iorga B., Adamek N., Geeves M.A. (2007). The slow skeletal muscle isoform of myosin shows kinetic features common to smooth and non-muscle myosins. J. Biol. Chem..

[10] Siemankowski R.F., White H.D. (1984). Kinetics of the interaction between actin, ADP, and cardiac myosin-S1. J. Biol. Chem..

[11] Flamig D.P., Cusanovich M.A. (1983). Kinetic comparison of normal and thyrotoxic bovine cardiac myosin subfragment-1. J. Biol. Chem..

[12] Smith S.J., Cusanovich M.A. (1984). Bovine cardiac myosin subfragment 1. Transient kinetics of ATP hydrolysis. J. Biol. Chem..

[13] Toniolo L., Maccatrozzo L., Patruno M., Caliaro F., Mascarello F., Reggiani C. (2005). Expression of eight distinct MHC isoforms in bovine striated muscles: evidence for MHC-2B presence only in extraocular muscles. J. Exp. Biol..

[14] Wank V., Fischer M.S., Walter B., Bauer R. (2006). Muscle growth and fiber type composition in hind limb muscles during postnatal development in pigs. Cells Tissues Organs.

[15] Toniolo L., Patruno M., Maccatrozzo L., Pellegrino M.A., Canepari M., Rossi R. (2004). Fast fibres in a large animal: fibre types, contractile properties and myosin expression in pig skeletal muscles. J. Exp. Biol..

[16] Iwamoto H., Oiwa K., Kovacs M., Sellers J.R., Suzuki T., Wakayama J.i. (2007). Diversity of structural behavior in vertebrate conventional myosins complexed with actin. J. Mol. Biol..

[17] Whittaker M., Wilson-Kubalek E.M., Smith J.E., Faust L., Milligan R.A., Sweeney H.L. (1995). A 35-Å movement of smooth muscle myosin on ADP release. Nature.

[18] Jontes J.D., Wilson-Kubalek E.M., Milligan R.A. (1995). A 32 degree tail swing in brush border myosin I on ADP release. Nature.

[19] Wells A.L., Lin A.W., Chen L.Q., Safer D., Cain S.M., Hasson T. (1999). Myosin VI is an actin-based motor that moves backwards. Nature.

[20] Millar N.C., Geeves M.A. (1988). Protein fluorescence changes associated with ATP and adenosine 5′-[gamma-thio]triphosphate binding to skeletal muscle myosin subfragment 1 and actomyosin subfragment 1. Biochem. J..

[21] Johnson K.A., Taylor E.W. (1978). Intermediate states of subfragment 1 and actosubfragment 1 ATPase: reevaluation of the mechanism. Biochemistry.

[22] Lymn R.W., Taylor E.W. (1971). Mechanism of adenosine triphosphate hydrolysis by actomyosin. Biochemistry.

[23] Bagshaw C.R., Trentham D.R. (1973). The reversibility of adenosine triphosphate cleavage by myosin. Biochem. J..

[24] Stein L.A., White M.P., Annis D.T. (1989). Biochemical kinetics of porcine cardiac subfragment-1. II. Pre-steady-state studies of the initial phosphate burst. Circ. Res..

[25] Cremo C.R., Geeves M.A. (1998). Interaction of actin and ADP with the head domain of smooth muscle myosin: implications for strain-dependent ADP release in smooth muscle. Biochemistry.

[26] Geeves M.A., Perreault-Micale C., Coluccio L.M. (2000). Kinetic analyses of a truncated mammalian myosin I suggest a novel isomerization event preceding nucleotide binding. J. Biol. Chem..

[27] Marston S.B., Taylor E.W. (1980). Comparison of the myosin and actomyosin ATPase mechanisms of the four types of vertebrate muscles. J. Mol. Biol..

[28] Nyitrai M., Geeves M. (2004). Adenosine diphosphate and strain sensitivity in myosin motors. Phil. Trans. Roy. Soc. ser. B.

[29] Veigel C., Coluccio L.M., Jontes J.D., Sparrow J.C., Milligan R.A., Molloy J.E. (1999). The motor protein myosin-I produces its working stroke in two steps. Nature.

[30] Lister I., Schmitz S., Walker M., Trinick J., Buss F., Veigel C., Kendrick-Jones J. (2004). A monomeric myosin VI with a large working stroke. EMBO J..

[31] Veigel C., Molloy J.E., Schmitz S., Kendrick-Jones J. (2003). Load-dependent kinetics of force production by smooth muscle myosin measured with optical tweezers. Nature Cell Biol..

[32] Veigel C., Wang F., Bartoo M.L., Sellers J.R., Molloy J.E. (2002). The gated gait of the processive molecular motor, myosin V. Nature Cell Biol..

[33] Capitanio M., Canepari M., Cacciafesta P., Lombardi V., Cicchi R., Maffei M. (2006). Two independent mechanical events in the interaction cycle of skeletal muscle myosin with actin. Proc. Natl Acad. Sci. USA.

[34] Metzger J.M. (1996). Effects of phosphate and ADP on shortening velocity during maximal and submaximal calcium activation of the thin filament in skeletal muscle fibers. Biophys. J..

[35] Wang G., Kawai M. (1996). Effects of MgATP and MgADP on the cross-bridge kinetics of rabbit soleus slow-twitch muscle fibers. Biophys. J..

[36] Karatzaferi C., Myburgh K.H., Chinn M.K., Franks-Skiba K., Cooke R. (2003). Effect of an ADP analog on isometric force and ATPase activity of active muscle fibers. Am. J. Physiol. Cell Physiol..

[37] Chase P.B., Kushmerick M.J. (1995). Effect of physiological ADP concentrations on contraction of single skinned fibers from rabbit fast and slow muscles. Am. J. Physiol. Cell Physiol..

[38] Nyitrai M., Rossi R., Adamek N., Pellegrino M.A., Bottinelli R., Geeves M.A. (2006). What limits the velocity of fast-skeletal muscle contraction in mammals?. J. Mol. Biol..

[39] Margossian S.S., Lowey S. (1982). Preparation of myosin and its subfragments from rabbit skeletal muscle. Methods Enzymol..

[40] Taylor R.S., Weeds A.G. (1976). The magnesium-ion-dependent adenosine triphosphatase of bovine cardiac myosin and its subfragment-1. Biochem. J..

[41] Pardee J.D., Spudich J.A. (1982). Purification of muscle actin. Methods Enzymol..

[42] Criddle A.H., Geeves M.A., Jeffries T. (1985). The use of actin labelled with N-(1-pyrenyl)iodoacetamide to study the interaction of actin with myosin subfragments and troponin/tropomyosin. Biochem. J..

[43] Bagshaw C.R., Eccleston J.F., Eckstein F., Goody R.S., Gutfreund H., Trentham D.R. (1974). The magnesium ion-dependent adenosine triphosphatase of myosin. Two-step processes of adenosine triphosphate association and adenosine diphosphate dissociation. Biochem. J..

[44] Clark R., Ansari M.A., Dash S., Geeves M.A., Coluccio L.M. (2005). Loop 1 of transducer region in mammalian class I myosin, Myo1b, modulates actin affinity, ATPase activity, and nucleotide access. J. Biol. Chem..

[45] Ritchie M.D., Geeves M.A., Woodward S.K., Manstein D.J. (1993). Kinetic characterization of a cytoplasmic myosin motor domain expressed in Dictyostelium discoideum. Proc. Natl. Acad. Sci. USA.

[46] Pellegrino M.A., Canepari M., Rossi R., D'Antona G., Reggiani C., Bottinelli R. (2003). Orthologous myosin isoforms and scaling of shortening velocity with body size in mouse, rat, rabbit and human muscles. J. Physiol..

